# Supplementary material for: Identification of photoperiod-induced specific miRNAs in the adrenal glands of Sunite sheep (Ovis aries)
Source: Front Vet Sci. 2022 Jul 22;9:888207. doi: 10.3389/fvets.2022.888207 (PMC9354845; doi:10.3389/fvets.2022.888207)
Supplement: Supplementary file 1 [file Data_Sheet_1.ZIP › Supplementary file/miRDeep2 file.zip!/miRDeep2 file/Novel_Structure/SP42A3/Novel_135.pdf]

Provisional ID	:	NC_019464.2_13636
Score total	:	1.5
Score for star read(s)	:	-1.3
Score for read counts	:	-0.3
Score for mfe	:	1.6
Score for randfold	:	1.6
Score for cons. seed	:	
Total read count	:	11
Mature read count	:	11
Loop read count	:	0
Star read count	:	0

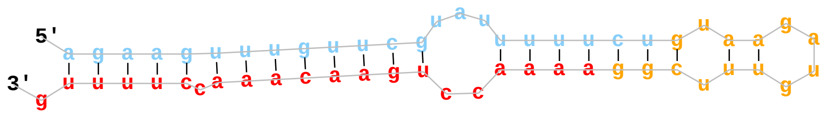

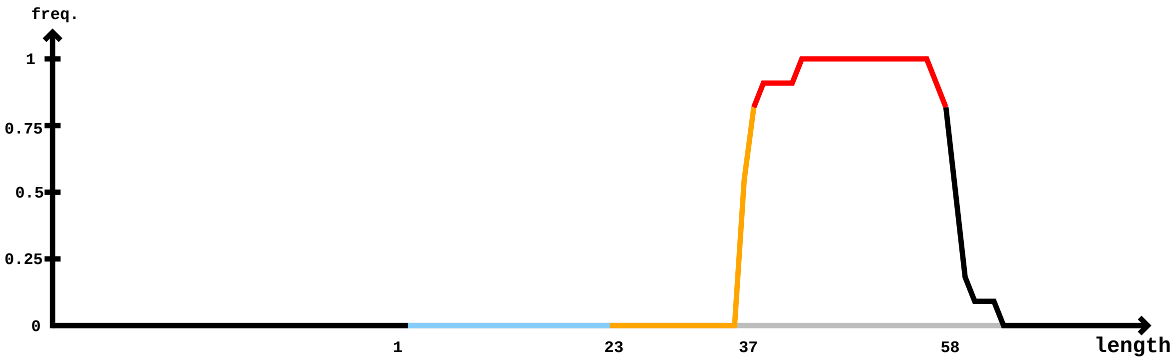


```
                                      Star                  Mature
5'- ugaguuuccuguaaacagaucauauugguuggccaagaaguuuguucguauuuuucuguaagauguuucggaaaaccugaacaaaccuuuuggccaauccaguacaagcaga   -3'    exp
    ........(((....)))....((((((((((((((((.(((((((((...(((((((.((....)).)))))))..))))))))).)))))))))).))))))........    reads    mm    sample
    ......................................................................gaaaaccugaacaaacUuuu......................    1        1     seq
    ......................................................................gaaaaccugaacaaacUuuuu.....................    1        1     seq
    ......................................................................gaaaaccugaacaaacUuuuug....................    3        1     seq
    ......................................................................gaaaaccugaacaaaccuuuug....................    1        0     seq
    .......................................................................aaaaccugaacaaacUuuuug....................    3        1     seq
    ........................................................................aaaUcugaacaaaccuuuuggc..................    1        1     seq
    ............................................................................cugaacaaaccuuuuggccaa...............    1        0     seq
```
